# Alpha-1 Antitrypsin PI M Heterozygotes with Rare Variants: Do They Need a Clinical and Functional Follow-Up?

**DOI:** 10.3390/jcm13041084

**Published:** 2024-02-14

**Authors:** Anna Annunziata, Giuseppe Fiorentino, Marco Balestrino, Roberto Rega, Sara Spinelli, Lidia Atripaldi, Alessio Sola, Federica Massaro, Cecilia Calabrese

**Affiliations:** 1 Department of Intensive Care, Azienda Ospedaliera di Rilievo Nazionale dei Colli, 80131 Naples, Italy; anna.annunziata@gmail.com (A.A.); giuseppefiorentino1@gmail.com (G.F.); robertorega09@gmail.com (R.R.);; 2 Department of Translational Medical Sciences, University of Campania “Luigi Vanvitelli”, Azienda Ospedaliera di Rilievo Nazionale dei Colli, 80131 Naples, Italy; marcobalestrino.mb@gmail.com (M.B.); lidia.atripaldi@virgilio.it (L.A.); alessio.sola.26.1224@gmail.com (A.S.); massaro.federica1993@gmail.com (F.M.)

**Keywords:** alpha-1 antitrypsin deficiency, rare allelic variants of gene SERPINA-1, PI*M heterozygotes, spirometry, diffusing capacity of the lung for carbon monoxide, functional follow-up

## Abstract

**(1) Background**: Few data are available on the risk of airway dysfunction in protease inhibitor (PI*) M heterozygotes carrying rare null or deficient allelic variants of the gene SERPINA-1 (PI*MR). **(2) Methods**: In this observational study, in a cohort of PI*MR heterozygotes, we evaluated respiratory functional parameters at baseline and at one-year follow-up. Moreover, we compared such parameters with those of the PI*MZ and PI*MS patients. **(3) Results**: A total of 60 patients were recruited; 35 PI*MR, 11 PI*MZ and 14 PI*MS. At the annual follow-up, the PI*MR and PI*MZ patients demonstrated a significantly higher FEV_1_ decline than the PI*MS group (*p* = 0.04 and *p* = 0.018, respectively). The PI*MR patients showed a significant increase in DLCO annual decline in comparison with the PI*MS group (*p* = 0.02). At baseline, the PI*MR smoking patients, compared with nonsmokers, showed statistically significant lower values of FEV_1_, FEV_1_/FVC and DLCO (*p* = 0.0004, *p* < 0.0001, *p* = 0.007, respectively) and, at the one-year follow-up, they displayed a significantly higher FEV_1_ and DLCO decline (*p* = 0.0022, *p* = 0.011, respectively). PI*MR heterozygotes with COPD showed a significantly higher FEV_1_, FEV_1_/FVC and DLCO annual decline in comparison with healthy PI*MR (*p* = 0.0083, *p* = 0.043, *p* = 0.041). **(4) Conclusions**: These results suggest that PI*MR heterozygotes, particularly smokers with COPD, have a greater annual decline in respiratory functional parameters and need to be monitored.

## 1. Introduction

Alpha-1 antitrypsin deficiency (AATD) is a clinically under-recognized autosomal, codominant, genetic disorder characterized by decreased serum levels and/or functionality of alpha-1 antitrypsin (AAT), which is a 152-kDa glycoprotein that represents the major inhibitor of the human neutrophil elastase. It is encoded by the highly pleomorphic gene SERPINA-1, with more than 500 allelic variants that have been identified and at least 60 predisposing to AATD lung, liver or other systemic diseases [1].

Based on protein isoelectric focusing, the “wild type” allelic variant is defined as protease inhibitor (PI*) M and it has an intermediate isoelectric point; the letters from A to L indicate the allelic variants with a position closer to the anode and the letters from N to Z are closer to the cathode.

The most common PI-deficient variants are the Z (Glu366Lys), which is associated with plasma levels approximately 10–15% of the normal M allele, and the S (Glu288Val), which is responsible for milder deficiency with plasma levels 50–60% of normal [2,3]. The null variants, termed Q0, are characterized by almost absent serum levels of AAT, while the AAT dysfunctional allelic variants are unable to exert the anti-protease function althoughtheir serum levels are in the normal range.

In addition, many other rare allelic variants have been described. They are identified by the alphabetical letter corresponding to the isoelectric point and the place of birth of the index case. However, the effects of many rare variants on a predisposition to AATD diseases are not well known.

The risk of lung disease is clearly established in patients carrying the PI null variants or the deficient genotypes PI*ZZ and PI*ZS, while it seems to be less relevant in PI*SS homozygotes. In these patients, the probability of developing chronic obstructive pulmonary disease (COPD), emphysema and/or bronchiectasis is greater in heavy smokers and a recent study has identified other risk factors, such as low body weight and older age [4]. On the contrary, the risk of lung damage and airway dysfunction displayed by the presence of only one Z, S, null or rare alleles in PI*M heterozygotes is more controversial, particularly in those carrying the rare variants.

Several studies have demonstrated that heavy-smoking PI*MZ heterozygotes compared with neversmoking PI*MM homozygotes have a lower lung function, an increased prevalence of COPD and a larger annual decline in the first second flow expiratory volume (FEV_1_) [5,6,7,8,9]. In contrast, a meta-analysis of 17 studies on PI*MS heterozygotes has shown that, despite reduced plasma AAT levels, these subjects are not at risk of airway obstruction, COPD or greater FEV_1_ decline [10]. However, a case report revealed an association between this genotype and the presence of lung bullae [11]. In asthmatic patients, heterozygosity for AAT with PI*MZ and PI*MS genotypes is associated with small airway dysfunction and with lung air trapping [12]. Few data are available on PI*M heterozygotes with null or rare allelic variants and whether they have to undergo a clinical and functional follow-up has not yet been established. For this reason, in the present study, in an aggregated cohort of PI*M heterozygotes with any rare or null variants (PI*MR), we evaluated the respiratory functional parameters at the baseline and at a one-year follow-up. Moreover, we compared such parameters with those of PI*MZ and PI*MS patients.

## 2. Materials and Methods

PI*MR, PI*MZ and PI*MS heterozygous patients were enrolled at the divisions of “Malattie, Fisiopatologia e RiabilitazioneRespiratoria” and “ClinicaPneumologica Luigi Vanvitelli”, Monaldi Hospital, Naples, Italy.

All PI*M heterozygotes were either patients complaining of respiratory symptoms such as a cough and breathlessness of unknown cause, or relatives of AATD patients with serum AAT levels < 120 mg/dL in the presence of normal blood C-reactive protein concentration. Those consenting to participate were enrolled in this study.

At baseline, the following data were collected, and these instrumental exams were performed:

- Demographic characteristics (age and sex);
- Smoking status (never, former or actual);
- Serum AAT levels, quantified by nephelometry (Atellica^®^Siemens);
- AAT immunophenotyping (SebiaHydrasys® System) and SERPINA-1 gene qualitative analysis (DNA IQ System, Promega or PAX gene Blood DNA kit, PreAnalytix or DNA Blood Mini kit, Qiagen), both performed in the same reference center;
- Spirometry, using the Vyntus BODY (Vyaire Medical), according to the American Thoracic Society/European Respiratory Society (ATS/ERS) guidelines [13]. The following parameters were measured: forced expiratory volume in the first second (FEV_1_), forced vital capacity (FVC) expressed as absolute values in litres (L) and percentages of the predicted value (%) and FEV_1_/FVC ratio;
- Diffusing capacity of the lung forcarbon monoxide (DLCO) by the single-breath test. All data were expressed as absolute values in mL.min^−1^.mmHg^−1^ and percentages of the predicted value (%), according to the ATS/ERS guidelines [14];
- High-resolution computed tomography (HRTC), to evaluate the presence of panlobular and centrilobular emphysema, bronchiectasis and/or other lung abnormalities.

On the basis of clinical symptoms, spirometric abnormalities and HRCT lung involvement, and in accordance with the 2023 GOLD recommendations [15], PI*MR, PI*MZ and PI*MS patients were considered if they were affected by the following:

- COPD, in the presence of symptoms of dyspnea, and/or chronic cough, and/or sputum production and a post-bronchodilator FEV_1_/FVC < 0.7, with or without CT evidence of emphysema or bronchiectasis;
- Pre-COPD, in the presence of respiratory symptoms, and/or other detectable structural and/or functional abnormalities, in the absence of airflow obstruction on forced spirometry. In the present study, all pre-COPD patients showed structural abnormalities on HRTC with or without associated respiratory symptoms.

All patients underwent an annual clinical and functional (spirometry and DLCO) follow-up.

Statistical analyses of the data were performed using GraphPad Prism 8.0 (GraphPad Software Inc., La Jolla, CA, USA). The “one-way” ANOVA test was used to analyze any statistically significant differences at baseline in age, serum AAT1 levels and spirometric values between the three groups. The conformity of the data to the normal distribution was tested with the Shapiro–Wilk test. Data that passed the normality test were analyzed with two-tail paired *t*-tests, otherwise a nonparametric Mann–Whitney U test was used to calculate statistical differences. Values of *p* < 0.05 were considered significant. Data are expressed as means ± standard deviation (SD).

## 3. Results

We recruited 60 PI*M heterozygous patients: 35 PI*MR, 11 PI*MZ and 14 PI*MS.

In the PI*MRgroup, the rare allelic variants were M_Lowell_ (9), M_Procida_ (8), M_Wurzburg_ (5), M_Whitstable_ (1), I (3), V (2), S_Munich_ (2), Q0_Oreum_ (4) and Q0_Perugia_ (1).

### 3.1. Baseline

The baseline characteristics of the study population are shown in Table 1.

**Figure 1:**
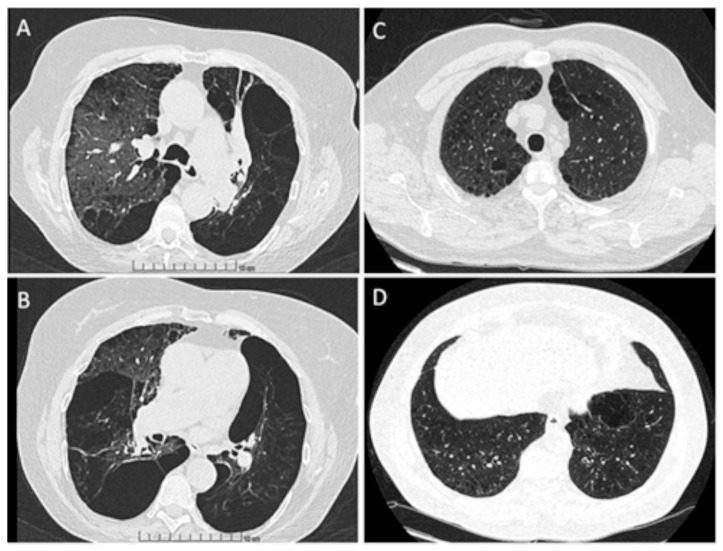
Chest high-resolution CT scan; axial view of upper lobes (**A**) and lower lobes (**B**) of lungs of an active smoking patient with M/MProcida genotype; axial view of upper lobes (**C**) and lower lobes (**D**) of lungs of a never smoker patient with M/I genotype.

Some examples of HRCT features of PI*M heterozygous patients are shown in Figure 1.

The prevalence of COPD, pre-COPD and healthy subjects in the PI*MR, PI*MZ and PI*MS groups with their respective age, smoking habits and presence of HRCT emphysema are described in Table 2.

**Figure 2:**
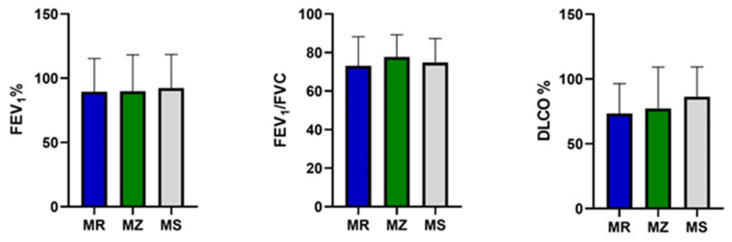
FEV_1_, FEV_1_/FVC and DLCO in the protease inhibitor (PI*) MZ, MS and MR groups. Abbreviations: FEV_1_, forced expiratory volume in the first second; FVC, forced vital capacity; DLCO, diffusing capacity of the lung for carbon monoxide.

At baseline, by comparing the three groups of PI*M heterozygotes, we did not find any statistically significant difference in age, sex and serum alpha-1 antitrypsin value (Table 1). In addition, no significant differences were detected in the spirometry (FEV_1_, FEV_1_/FVC) and DLCO values between the three groups of patients (Figure 2).

### 3.2. One-Year Follow-Up

At the annual follow-up, PI*MR and PI*MZ patients demonstrated a significant increase in FEV_1_ decline (L) in comparison with the PI*MS group (−0.136 ± 0.15 vs. −0.032 ± 0.21; *p* = 0.04; −0.174 ± 0.1 vs. −0.032 ± 0.2; *p* = 0.018, respectively), without any statistically significant difference between PI*MR and PI*MZ groups (−0.136 ± 0.15 vs. −0.174 ± 0.1; *p* = 0.12).

In addition, PI*MR patients demonstrated an increase in DLCO annual decline (mL.min^−1^.mmHg^−1^) in comparison with the PI*MS group (−0.69 ± 0.80 vs. −0.18 ± 0.54; *p* = 0.02), while there were no significant differences between PI*MR and PI*MZ patients (−0.69 ± 0.80 vs. −0.28 ± 0.36; *p* = 0.07) and between PI*MZ and PI*MS patients (−0.28 ± 0.36 vs. −0.18 ± 0.54; *p* = 0.44).

**Figure 3:**
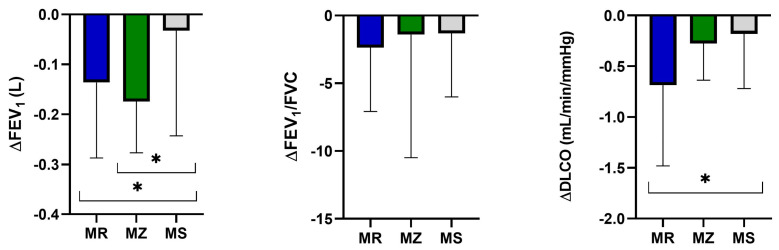
Annual FEV_1_, FEV_1_/FVC and DLCO decline in the protease inhibitor (PI*) MZ, MS and MR groups. Abbreviations: ∆, decline; FEV_1_, forced expiratory volume in the first second; FVC, forced vital capacity; DLCO, diffusing capacity of the lung for carbon monoxide; *, *p*-value < 0.05.

In contrast, the FEV_1_/FVC ratio did not decline differently in the three PI*M heterozygous groups (Figure 3).

### 3.3. PI*MR Group

**Figure 4:**
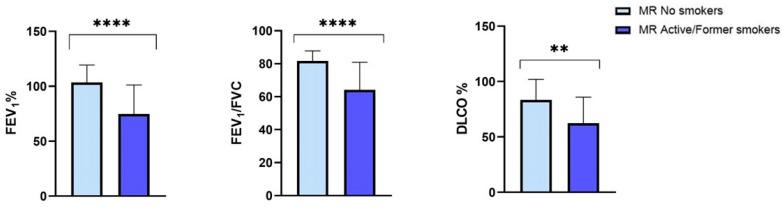
Baseline FEV_1_, FEV_1_/FVC and DLCO in the protease inhibitor (PI*) MR smoking and nonsmoking subgroups. Abbreviations: FEV_1_, forced expiratory volume in the first second; FVC, forced vital capacity; DLCO, diffusing capacityof the lung forcarbon monoxide; **, *p*-value < 0.01; ****, *p*-value < 0.0001.

Focusing on the larger PI*MR group, we observed that, at the baseline, smoking patients, compared with the nonsmokers, showed statistically significant lower percentage values of FEV_1_ (74.64 ± 26.52 vs. 103.44 ± 18.8, *p* = 0.0004), FEV_1_/FVC (64.10 ± 16.76 vs. 81.67 ± 6.15, *p* < 0.0001) and DLCO (62.37 ± 23.58 vs. 83.47 ± 18.41, *p* = 0.007) (Figure 4).

**Figure 5:**
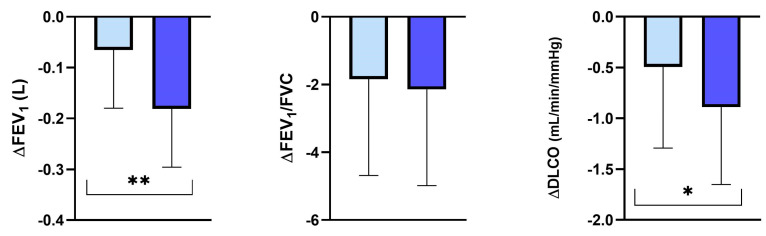
FEV_1_, FEV_1_/FVC and DLCO decline in the protease inhibitor (PI*) MR smoking and nonsmoking subgroups. Abbreviations: FEV_1_, forced expiratory volume in the first second; FVC, forced vital capacity; DLCO, diffusing capacity of the lung for carbon monoxide; *, *p*-value < 0.05; **, *p*-value < 0.01.

In addition, at the annual follow-up, PI*MR smokers showed a significantly higher FEV_1_ and DLCO annual decline (L) in comparison with the nonsmoking patients (−0.20 ± 0.11 vs. −0.07 ± 0.11; *p* = 0.0022; −0.89 ± 0.80 vs. −0.49 ± 0.76; *p* = 0.011, respectively). On the contrary, there was no significant difference in the FEV_1_/FVC annual decline between PI*MR smokers and nonsmokers (−2.14 ± 2.84 vs. −1.84 ± 2.84, *p* = 0.32) (Figure 5).

When we divided PI*MR patients in relation to their respiratory disease status, COPD patients showed statistically significant lower FEV_1_% and FEV_1_/FVC values than pre-COPD patients, who, in turn, demonstrated significantly decreased FEV_1_% and DLCO % values in comparison with healthy subjects. All the functional respiratory parameters were significantly lower in COPD patients in comparison with healthy subjects (Table 3).

**Figure 6:**
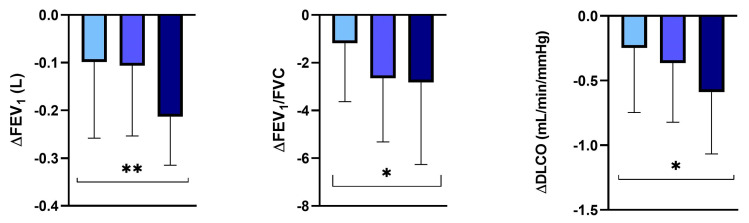
FEV_1_, FEV_1_/FVC and DLCO mean decline in the PI*MR healthy, pre-COPD and COPD subgroups. Abbreviations: ∆, decline; FEV_1_, forced expiratory volume in the first second; FVC, forced vital capacity; DLCO, diffusing capacity of the lung for carbon monoxide; (* *p*-value < 0.05; ** *p*-value < 0.01).

At the annual follow-up, PI*MR COPD patients showed a significantly higher FEV_1_, FEV_1_/FVC and DLCO annual decline in comparison with healthy PI*MR heterozygotes (−0.213 ± vs. −0.098; *p* = 0.0083; −2.82 ± 3.44 vs.−1.185 ± 2.44; *p* = 0.043; −0.589 ± 0.726 vs. −0.246 ± 0.497, *p* = 0.041), while the difference between PI*MR COPD and pre-COPD and between pre-COPD and healthy subjects in their annual decline in FEV_1_, FEV_1_/FVC and DLCO did not reach statistical significance (Figure 6).

The FEV_1_ and DLCO annual decline and the AAT serum levels displayed by each PI*MR subgroup, identified on the basis of the rare or null allelic variants, are shown in Supplementary Table S1.

## 4. Discussion

In the present study, we demonstrated, for the first time, a significant increase in the annual decline in FEV_1_ and DLCO in a cohort of PI*M heterozygotes with any null or rare deficient allelic variants in comparison with the PI*MS heterozygotes. We found no significant difference with the PI*MZ heterozygotes. Cigarette smoking represents an important risk factor for the PI*MR heterozygotes, as demonstrated by the larger FEV_1_, FEV_1_/FVC and DLCO yearly drop observed in smoking subjects compared to nonsmoking subjects. Once PI*MR heterozygotes have developed COPD, they display a greater annual decline in all the main respiratory functional parameters in comparison with both the pre-COPD and healthy subjects. These results suggest that PI*M heterozygotes who carry one rare or null allelic variant need attention and need to be monitored in a similar way to PI*MZ heterozygotes.

PI*MR subjects must quit their cigarette smoking habit. In fact, although COPD patients were older than the healthy subjects, the difference in the mean age was not significant. On the contrary, the cigarette smoking habit was significantly more prevalent in the COPD subgroup. Lifestyle changes have been strongly suggested for the PI*MZ heterozygotes. In fact, recent studies in the literature have clearly demonstrated that subjects carrying the PI*MZ genotypes have an increased risk of developing lung disease if exposed to smoking or other airborne or industrial pollutants. These data are particularly relevant considering the prevalence of this genotype, with more than 35 million PI*MZ heterozygotes identified worldwide [8,16,17]. As the PI*MZ genotype is a relatively common risk factor for COPD, lifestyle modifications can substantially influence prevention of the disease. Indeed, data regarding the prevalence of PI*M heterozygotes with any null or rare deficient allelic variants are scarcely reported [18,19].

Regarding the need for a regular clinical follow-up, it is actually suggested for the PI* MZ heterozygotes affected by COPD. As shown by the results of our study, PI* MR heterozygotes affected by COPD display a greater annual decline when compared not only with healthy subjects, but also with pre-COPD subjects.

The therapeutic management of patients carrying the PI*MZ genotype, characterized by a mild or moderate AAT deficiency, is not yet defined. In fact, in contrast with the most common severe deficient genotype PI Z/Z, debate still exists on the opportunity of AAT augmentation therapy in PI*MZ individuals with lung disease. Without any clear evidence in the literature on the clinical benefits of AAT therapy in PI*MZ individuals, current guidelines do not support it [20]. The data in the literature regarding PI*M heterozygotes with any rare or null allelic variants are very scarce, so the clinical management of these patients is much more controversial than that of PI*MZ heterozygotes. There are only a few case studies describing the treatment of heterozygous patients with deficient variants [20,21,22].

However, the recent implementation of serum AAT dosage and SERPINA-1 genetic testing in patients affected by several respiratory diseases or unexplained dyspnea has increased the detection not only of the already known deficient variants, but also of the new rare PI* alleles [16,23]. We suppose that their number will increase further, as the most recent AATD position statement of the Thoracic Society of Australia and New Zealand has expanded the indication of AATD tests in all patients with chronic airflow obstruction, in asthma patients with persistent airflow limitation, in subjects with emphysema disproportionate to their smoking history or in the presence of liver or skin disease [24]. In addition, the availability of a genotyping test that can be performed not only on dried blood spot samples but also on buccal swabs may implement both the diagnosis of AATD and the identification of new rare PI* allelic variants [25,26].

Actually, only a few descriptive studies on PI*M heterozygotes carrying rare deficient or null variants are available in the literature. Ferrarotti et al. described eight PI*MQ0 subjects who were affected by emphysema, asthma orchronic bronchitis, independent of their smoking habit [27]. Papiris et al. identified seven nonsmoking PI*M heterozygotes with rare deficient or null variants among 45 Greek adults with early-onset pulmonary emphysema and low serum AAT levels [28]. Finally, Ortega et al., in a cohort of heavy smokers with COPD drawn from the SPIROMICS study, observed that every PI*M heterozygosity for each Z, S or rare allele variant was associated with increased CT scan-based emphysema and functional small-airway diseases. In particular, the authors described 19 heterozygotes carrying at least 1 rare allelic variant associated with low serum AAT levels and cumulatively increased CT emphysema [16]. We strongly advocate forstudies that monitor clinical, functional and radiological parameters of PI*MR heterozygotes over time.

The present study has three main limitations. Firstly, we aggregated in a single group PIM* heterozygotes carrying any rare allelic variants. This was due to the difficulty of testing, with sufficient power, the effect of each PI* rare variant on lung function. Secondly, in the PI*MR group, we aggregated subjects with null and deficient allelic variants, even though the Q0* gene could further impair the AAT serum levels and the susceptibility to lung disease; however, this event did not occur in our cohort of PI*MR heterozygotes, as shown in Table 3. Thirdly, the percentage of patients affected by COPD is lower in the PI* MZ group compared to the PI*MS and PI*MR groups. This may be due to the attention we paid to these patients, encouraging them to stop cigarette smoking and, in this way, preventing the development of COPD.

## 5. Conclusions

The results of the present study demonstrate that PI*MR heterozygotes have a greater annual decline in respiratory functional parameters in comparison with PI*MS heterozygotes, similar to PI*MZ subjects. In particular, PI*MR heterozygotes who smoke and those affected by COPD show the greatest impairment. These results suggest the need for early and regular clinical and pulmonary functional monitoring of PI*MR heterozygotes.

## Figures and Tables

**Table 1:** Baseline characteristics of PI*MR, PI*MZ and PI*MS groups. Abbreviations: FEV_1_, forced expiratory volume in the first second; FVC, forced vital capacity; DLCO, diffusing capacity of the lung for carbon monoxide; HRTC, high-resolution computed tomography; na, not applicable.

	PI*MR (*n* = 35)	PI*MZ (*n* = 11)	PI*MS (*n* = 14)	*p*
Age, yr, mean (SD)	52.6 (15.9)	60.5 (17.6)	58.6 (9.7)	0.23
Sex, F *n* (%)	19 (52.77)	2 (18.2)	8 (57.1)	na
Alpha-1Antitrypsin mg/dL mean (SD)	90.7 (17.6)	92.9 (15.6)	93.9 (14.4)	0.81
Actual and former smokers, *n* (%)	17 (48.6)	6 (54.5)	5 (35.7)	na
FEV_1_, % predicted mean (SD)	89.5 (25.9)	89.8 (28.4)	92.5 (26.1)	0.94
FEV_1_/FVC, mean (SD)	73.1 (15.1)	77.7 (11.5)	74.8 (12.6)	0.63
DLCO, % predicted mean (SD)	73.5 (23)	77.3 (31.9)	86.3 (23)	0.28
HRTC-defined Emphysema, *n* (%)	18 (51.4)	10 (90.9)	10 (71.4)	na

**Table 2:** Prevalence of COPD, pre-COPD and healthy subjects in the PI*MR, PI*MZ and PI*MS groups and their respective smoking habits and HRCT emphysema. Abbreviations: COPD, chronic obstructive pulmonary disease; HRTC, high-resolution computed tomography.

PI*	Parameters	COPD	PRE-COPD	Healthy
PI*MR (*n* = 35)	*n* (%) age, yr, mean (SD)	10 (28.6%) 60 (±13.3)	8 (22.9%) 56 (±11.5)	17 (48.5%) 47 (±14)
	Smokers, *n* (%)	10 (100%)	4 (50%)	3 (17.64%)
	HRCT Emphysema, *n* (%)	10 (100%)	8 (100%)	0 (0%)
PI*MZ (*n* = 11)	*n* (%) age, yr, mean (SD)	1 (9.1%) 73	9 (81.8%) 62 (±16.2)	1 (9.1%) 32
	Smokers *n* (%)	1(100%)	5 (55.5%)	0 (0%)
	HRCT Emphysema, *n* (%)	1 (100%)	9 (100%)	0 (0%)
PI*MS (*n* = 14)	*n* (%) age, yr, mean (SD)	3 (21.4%) 59 (±1.15)	5 (35.7%) 61 (±2.6)	6 (42.8%) 57 (±15)
	Smokers *n* (%)	3 (100%)	1 (20%)	1 (16.6%)
	HRCT Emphysema, *n* (%)	2 (66%)	5 (100%)	0 (0%)

**Table 3:** Functional parameters of COPD, pre-COPD and healthy protease inhibitor (PI*) MR heterozygotes. Abbreviations: COPD, chronic obstructive pulmonary disease; FEV_1_, forced expiratory volume in the first second; FVC, forced vital capacity; DLCO, diffusing capacity of the lung for carbon monoxide; ns, not significant.

Parameters	PI*MR COPD (10)	PI*MR PRE-COPD (8)	PI*MR Healthy (17)	COPD vs. PRE-COPD *p*	PRE-COPD vs. Healthy *p*	COPD vs. Healthy *p*
FEV_1_ (%)	62 ± 27	92.7 ± 10.6	104.1 ± 16.1	0.008	0.043	<0.0001
FEV_1_/FVC	53.5 ± 13.7	81 ± 3.1	81 ± 6.6	<0.0001	ns	<0.0001
DLCO (%)	57.1 ± 22.7	66.6 ± 19.7	87.5 ± 17	ns	0.014	0.0009

## Data Availability

The dataset used for the data analysis is available on reasonable request to the Corresponding Author.

## Supplementary Materials

The following supporting information can be downloaded at: https://www.mdpi.com/article/10.3390/jcm13041084/s1, Table S1: Alleles identified in the PI* MR group with their respective serum AAT values, FEV1 and DLCO decline. Abbreviations: AAT, alpha -1 antitrypsin; FEV1, forced expiratory volume in the first second; DLCO, diffusing capacity of the lung for carbon monoxide.
