# Supplementary figures and images for: Alpha-1 Antitrypsin PI M Heterozygotes with Rare Variants: Do They Need a Clinical and Functional Follow-Up?
Source: J Clin Med. 2024 Feb 14;13(4):1084. doi: 10.3390/jcm13041084 (PMC10889345; doi:10.3390/jcm13041084)

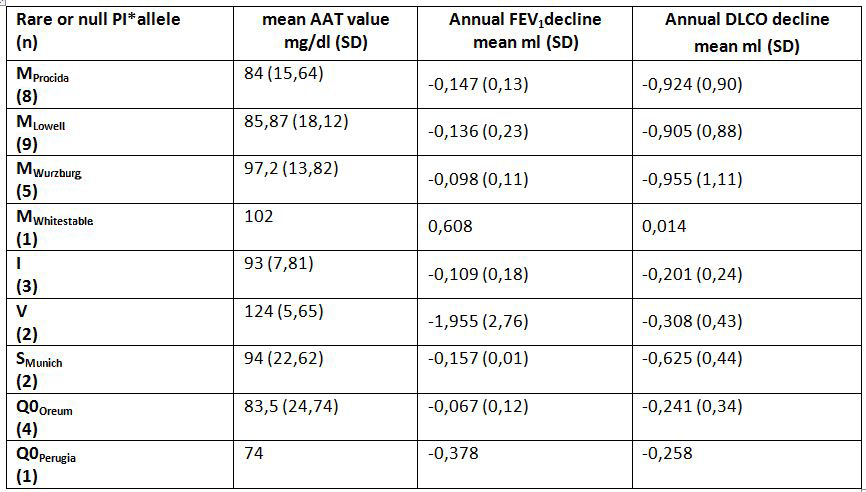

Supplement: Supplementary file 1 [file jcm-13-01084-s001.zip › Table S1.png]
